# An Inverse Class-E Power Amplifier for Ultrasound Transducer

**DOI:** 10.3390/s23073466

**Published:** 2023-03-26

**Authors:** Hojong Choi

**Affiliations:** Department of Electronic Engineering, Gachon University, Seongnam-daero 1342, Sujeong-gu, Seongnam 13120, Gyeonggi-do, Republic of Korea; hojongch@gachon.ac.kr; Tel.: +82-31-750-5591

**Keywords:** inverse Class-E power amplifier, ultrasound system, ultrasound transducer

## Abstract

An inverse Class-E power amplifier was designed for an ultrasound transducer. The proposed inverse Class-E power amplifier can be useful because of the low series inductance values used in the output matching network that helps to reduce signal distortions. Therefore, a newly designed Class-E power amplifier can obtain a proper echo signal quality. The measured output voltage, voltage gain, voltage gain difference, and power efficiency were 50.1 V, 22.871 dB, 0.932 dB, and 55.342%, respectively. This low voltage difference and relatively high efficiency could verify the capability of the ultrasound transducer. The pulse-echo response experiment using an ultrasound transducer was performed to verify the capability of the proposed inverse Class-E power amplifier. The obtained echo signal amplitude and pulse width were 6.01 mV_p-p_ and 0.81 μs, respectively. The −6 dB bandwidth and center frequencies of the echo signal were 27.25 and 9.82 MHz, respectively. Consequently, the designed Class-E power amplifier did not significantly alter the performance of the center frequency of the ultrasound transducer; therefore, it could be employed particularly in certain ultrasound applications that require high linearity and reasonable power efficiency.

## 1. Introduction

An ultrasound system comprises a transducer, transmitter, and receiver [1,2,3]. In the transmitter, a power amplifier is located in one of the last-stage electronics that controls the transducer’s performance [4,5,6,7]. Therefore, the transducer’s performance is relatively sensitive to the performance of the power amplifier [8,9,10,11,12]. The design of the power amplifier could consider this type of loading effect; therefore, many types of power amplifiers have been developed. Typical performance parameters of power amplifiers are gain, bandwidth, and linearity [13,14,15,16]. However, the best values for all parameters cannot be simultaneously achieved because the main electronic component is the transistor, which has unwanted parasitic resistance, capacitance, and inductance [17,18,19]. Therefore, a power amplifier must be specifically designed for various ultrasound applications.

We describe, here, the commercial power amplifiers for reference to previous research on ultrasound stimulation, neuromodulation, and ultrasound therapy applications. In order to generate 2.5 MHz and 50 V_p-p_, a power amplifier was used [20]. A waveform of 3.3 MHz and 0.4 V_p-p_ from a power amplifier was used to trigger a transducer [21]. A pulsed waveform of 46 MHz and 12 V_p-p_, generated using a power amplifier, was used [22]. In another study, a pulse waveform of 30 MHz and 20 V_p-p_ was generated using an amplifier [23]. A power amplifier that was used in ultrasound neuromodulation applications was employed to produce a pulsed sinusoidal waveform of 2 MHz. In addition, an impedance-matching circuit was useful for providing an effective stimulus [24,25]. A commercial power amplifier was used to produce 0.5 MHz and 100-cycle pulses. However, such power amplifiers exhibit long ring-down signals after the amplification [26,27]. A linear voltage amplifier with an output power of 9.43 mW and an operating frequency of 3.6 MHz was used [28]. The characteristics of the cells were analyzed using acoustic tweezers [29,30]. For the acoustic tweezers, a 50 MHz ultrasound transducer was triggered by a power amplifier [29].

In another study, a 50 MHz ultrasound transducer was used to simulate breast cancer cells [31]. However, an unmatched electrical impedance could cause great attenuation at higher frequencies [32]. A 50 mV_p-p_ input signal with a power amplifier was used to trigger the ultrasound transducer [33]. A 47 MHz transducer was triggered by a power amplifier with 18 V_p-p_ [34]. Therefore, previous studies have described that linear signal generation from a power amplifier could be desirable to achieve effective acoustic stimulation, neuromodulation, and ultrasound therapy performance.

Power amplifier classes are typically divided into linear and nonlinear power amplifiers [35]. Linear power amplifiers are classified as Class-A, Class-B, and Class-AB, and nonlinear power amplifiers are classified as Class-C, Class-D, Class-DE, and Class-E [36,37]. The main component of power amplifiers is the transistor. Furthermore, the operating mechanism of a power amplifier is related to the conduction angle of the transistor [38,39]. The full cycle of the conduction angle does not affect signal distortions; however, the low cycle of the conduction angle affects signal distortions, resulting in a low direct current (DC) power consumption [40]. Linear power amplifiers have low signal distortions and nonlinear power amplifiers have low power consumption with high signal distortions [41]. Moreover, nonlinear power amplifiers are used in wireless communication systems, whereas linear power amplifiers are used in wired communication systems [42,43,44,45].

**Figure 1:**
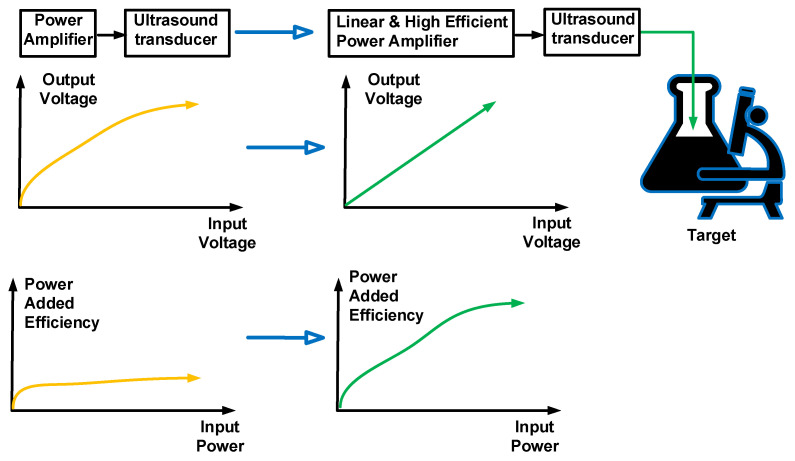
Concept of a Class-E power amplifier for ultrasound transducer.

This study presents the design of an inverse Class-E power amplifier, which could be useful for ultrasound transducers. The performance of the power amplifier could slightly affect the performance of the ultrasound transducer, owing to the characteristics of the inverse Class-E power amplifier. However, typical inverse Class-E power amplifiers with high signal distortions have been used in low-power communication systems [46]. Ultrasound systems typically work with low signal distortions at high voltages [47,48,49,50]. In particular, high linearity for acoustic stimulation applications is desirable. Thus, linear and relatively high-efficiency power amplifiers can be applied to ultrasound targets, as described in Figure 1. The inverse Class-E power amplifier was first applied to ultrasound transducer applications.

The remainder of this paper is organized as follows. Section 2 describes previous studies on power amplifiers for ultrasound applications. Section 3 describes the design and analysis of the inverse Class-E power amplifier. Section 4 presents the experimental results of the inverse Class-E power amplifier, such as the gain, bandwidth, and power-added efficiency with pulse-echo responses. Section 5 draws the conclusions and future work on the designed inverse Class-E power amplifier.

## 2. Studies on Ultrasound Applications

The existing designed power amplifiers for various ultrasound applications comprised Class-A, Class-AB, Class-B, Class-D, Class-DE, and Class-E types. A Class-A power amplifier with a voltage gain of 42 dB and an output voltage of 60 V_p-p_ was designed [51]. A Class-B power amplifier with a maximum output voltage of 90 V_p-p_, voltage gain of 40.9 dB, and bandwidth of 6.5 MHz was introduced [52]. A digital-to-analog converter and the digital pre-distortion technique were used to develop a Class-AB power amplifier with a second harmonic distortion (HD2) of −50 dB and a power efficiency of 30% [53]. Moreover, a Class-AB power amplifier with a center frequency of 20.046 kHz was used to drive a transducer [54]. In another study, a Class-AB power amplifier with a bandwidth of 15 MHz and an output voltage of 60 V was designed [55]. A Class-D power amplifier with a maximum output power of 2 kW and a bandwidth of 100 kHz was used [56]. A Class-D power amplifier with a bandwidth of 3.6 kHz, output voltage of 125 V, and efficiency of 95% was designed [57]. A Class-DE power amplifier with an output power of 800 mW and a bandwidth of 1010 kHz was developed to drive a magnetic resonance imaging (MRI)-integrated high-intensity focused ultrasound transducer [58]. A Class-E power amplifier with an output power of 133.3 mW and a center frequency of 41.27 kHz was designed [59]. A Class-E power amplifier with an operating frequency of 41.27 kHz and 39.7 W of power was designed [60]. These power amplifiers were designed for ultrasound transducers or imaging applications. However, no inverse Class-E power amplifier design has been reported for ultrasound transducers.

## 3. Materials and Methods

### 3.1. Description of the Inverse Class-E Power Amplifier

**Figure 2:**
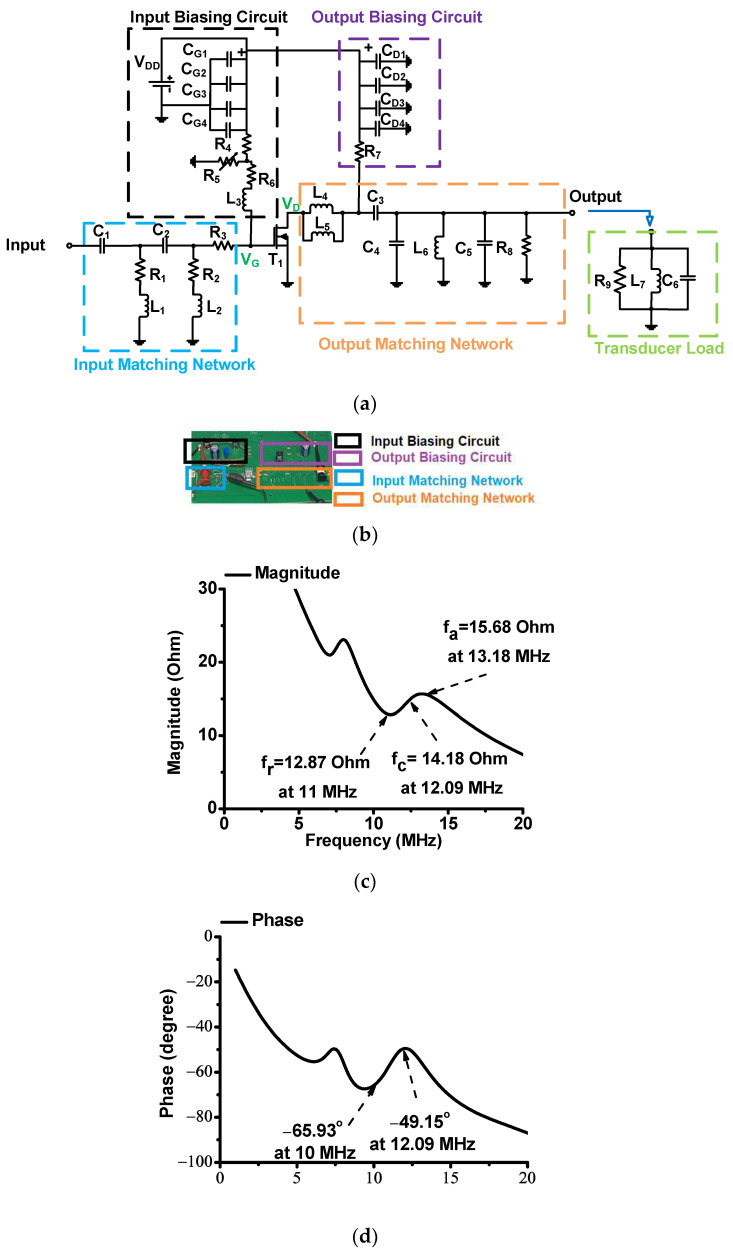
(**a**) Schematic diagram and (**b**) printed circuit board of the designed inverse Class-E power amplifier. (**c**) The magnitude and (**d**) phase of the impedance of the ultrasound transducer.

Figure 2a shows a schematic diagram of the designed inverse Class-E power amplifier. An inverse Class-E power amplifier was designed for ultrasound applications. Unfortunately, a similar inverse Class-E power amplifier design used in the communication system cannot be used; therefore, a new inverse Class-E power amplifier had to be redesigned. In the power amplifier, the component values were adjusted after obtaining a theoretical analysis because the theoretical inverse Class-E power amplifier had high efficiency and relatively low linearity. In order to achieve a highly linear output from the designed inverse Class-E power amplifier, a new design structure with additional adjustments was necessary to trade off the performance between the power efficiency and linearity.

In the input biasing circuit, an electrostatic capacitor (C_G1_ = 10 μF; Panasonic Corp., Newark, NJ, USA) and three additional capacitors (C_G2_ = 0.1 μF, C_G3_ = 1000 pF, C_G4_ = 47 pF; KEMET Corp., Simpsonville, CA, USA) were used [61]. A variable resistor (R_5_; KEMET Corp.) fed the gate bias DC voltage (V_G_) through a fixed resistor (R_6_) and inductor (1 μH, Coilcraft Inc., Silver Lake Road, IL, USA) to the main transistor (T_1_). In the output biasing circuit, an electrostatic capacitor (C_D1_ = 220 μF; Panasonic Corp., Newark, NJ, USA) and three additional capacitors (C_G2_ = 0.1 μF, C_G3_ = 1000 pF, C_G4_ = 47 pF; KEMET Corp., Simpsonville, CA, USA) were used [62]. A fixed resistor (R_7_) fed a drain-bias DC voltage (V_D_) to the main transistor. In the proposed inverse Class-E power amplifier, a choke inductor was used to feed a drain-bias DC voltage (V_D_) to the main transistor (T_1_); thus, the power amplifier could maximize the output power [63,64,65]. However, acoustic stimulation applications must be utilized by a power amplifier with reasonable linearity. Moreover, a fixed resistor (R_7_) was used instead of a choke inductor.

The input matching network was composed of two capacitors (C_1_ and C_2_; Vishay Intertechnology Inc., Malvern, PA, USA), three resistors (R_1_, R_2_, and R_3_; Vishay Siliconix, Siler Lake Road, IL, USA), and two inductors (L_1_ and L_2_; Coilcraft Inc., Silver Lake Road, IL, USA). In the output matching network, two inductors (L_4_ and L_5_; Coilcraft Inc.) with three capacitors (C_3_, C_4_, and C_5_; Vishay Siliconix), along with one inductor (L_6_; Coilcraft Inc.) and one resistor (R_8_; Vishay Siliconix), were utilized.

Figure 2b shows an inverse Class-E power amplifier developed on a fabricated two-layer printed circuit board. The input and output biasing circuits and input and output matching networks are indicated with different colors. The main transistor of the power amplifier was a high-voltage transistor (T_1_, ST Microlectronics Inc., Geneva, Switzerland). A heat sink with a square size of 1 cm^2^ was placed on top of the main transistor to reduce the side effects caused by heat [66]. The ultrasound transducer has an equivalent circuit model comprising parallel resistance, inductance, and capacitance [67,68]. Figure 2c shows the magnitude of the impedance of the ultrasound transducer. The magnitudes of the impedance are 14.92 Ω at 10 MHz and 14.18 Ω at 12.09 MHz, respectively. The magnitude variation between 10 MHz and the center frequency (12.09 MHz) is 0.74 Ω. Figure 2d shows the phase of the impedance of the ultrasound transducer. The phase angles of the impedances are −65.93° at 10 MHz and −49.15° at 12.09 MHz. The phase variation between 10 MHz and 12.09 MHz is 16.78°.

### 3.2. Operating Mechanism of the Inverse Class-E Power Amplifier

First, the input and output matching networks of the power amplifier must be obtained because the output voltage and voltage gain are related to these parameters [69].

**Figure 3:**
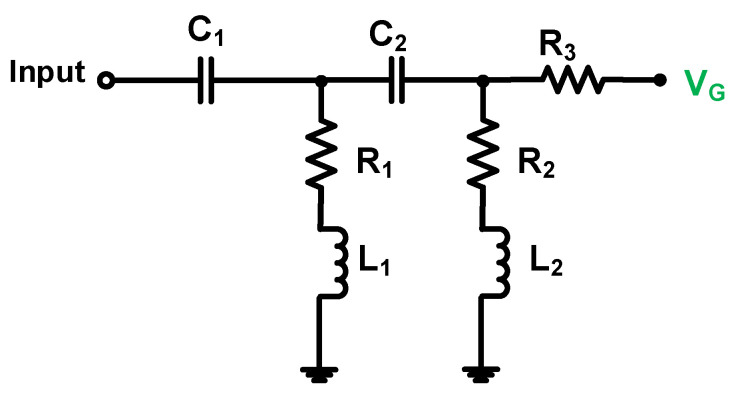
Schematic diagram of the input matching network.

As shown in Figure 3, the input matching network is a type of high-pass filter network. In the input matching network, the capacitance (C_1_) is serially connected to the resistance (R_1_) and inductance (L_1_) and is parallel to the capacitance (C_2_). In addition, the capacitance (C_2_) is serially connected to the resistance (R_2_) and inductance (L_2_) and is parallel to the resistance (R_3_). The resistances of R_1_ and R_2,_ with a lossless network comprising inductance and capacitance components, could help match the 50-Ω input impedance. Therefore, the input impedance (Z_in_) of the input matching network can be represented as follows:

(1) $$Z_{\mathrm{in}}=\left[\frac{1}{j2{\pi f}_{c}C_{1}}+\left(j2{\pi f}_{c}L_{1}+R_{1}\right)∥\frac{1}{j2{\pi f}_{c}C_{2}}\right]+\left[\left(j2{\pi f}_{c}L_{2}+R_{2}\right)∥R_{3}\right].$$

**Figure 4:**
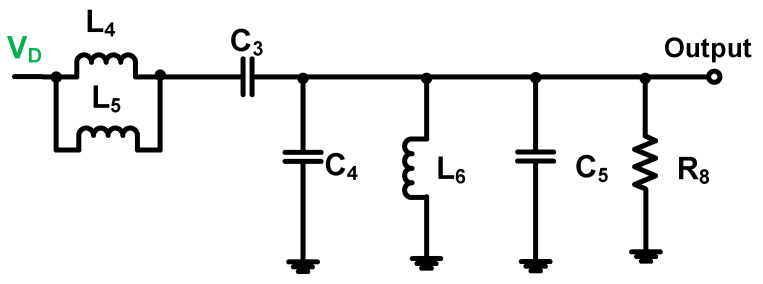
Schematic of the output matching network.

As shown in Figure 4, the output matching network is a low-pass filter network. Moreover, the inductances (L_4_ and L_5_) and the capacitance (C_3_) are parallel to the capacitances (C_4_ and C_5_), the inductance (L_6_), and the resistance (R_8_). Therefore, the output impedance (Z_out_) of the output matching network can be represented as follows:

(2) $$Z_{\mathrm{out}}=\left[\left(j2{\pi f}_{c}L_{1}\right)∥\left(j2{\pi f}_{c}L_{2}\right)+\frac{1}{j2{\pi f}_{c}C_{1}}\right]+\left[\frac{1}{j2{\pi f}_{c}\left(C_{4}+C_{5}\right)}∥\left(j2{\pi f}_{c}L_{6}\right)∥R_{8}\right].$$

The transfer functions of the power amplifier’s input and output frequencies (f_in_ and f_out_) can be obtained from the main transistor’s input and output gates and source and drain nodes [70,71,72]. Therefore, the input and output frequencies of the inverse Class-E power amplifier are expressed as follows:

(3) $$f_{\mathrm{in}}=[j2{\pi Z}_{\mathrm{in}}{\left(C_{\mathrm{gs}}+\left(1+g_{m}Z_{\mathrm{out}}\right)C_{\mathrm{gd}}\right]}^{-1},$$

(4) $$f_{\mathrm{out}}=[j2{\pi Z}_{\mathrm{out}}{\left(C_{\mathrm{ds}}+C_{\mathrm{gd}})\right]}^{-1},$$

where C_gs_, C_gd_, C_ds_, and g_m_ are the gate-source, gate-drain, and drain-source capacitances and transconductance of the main transistor (T_1_), respectively.

The designed power amplifier is a common-source topology-driven amplifier [73,74]. Therefore, the output voltage and output power of the inverse Class-E power amplifier can be obtained as follows:

(5) $$V_{\mathrm{out}}=-\frac{g_{m}Z_{\mathrm{out}}}{\left(1+\frac{f_{c}}{f_{\mathrm{in}}}\right)\left(1+\frac{f_{c}}{f_{\mathrm{out}}}\right)}V_{\mathrm{in}},$$

(6) $$P_{\mathrm{out}}={\left(\frac{g_{m}Z_{\mathrm{out}}}{\left(1+\frac{f_{c}}{f_{\mathrm{in}}}\right)\left(1+\frac{f_{c}}{f_{\mathrm{out}}}\right)}\right)}^{2}P_{\mathrm{in}},$$

where V_out_, P_in_, and P_out_ are the output voltage, input power, and output power of the inverse Class-E power amplifier, respectively.

In an inverse Class-E power amplifier, the theoretical values of the output matching circuit can be calculated as follows: The inverse Class-E power amplifier has particular characteristics owing to the operating mechanism of the output matching network. As shown in Figure 4, two inductances (L_4_ and L_5_) were selected to avoid the breakdown of the transistor owing to a high current. In addition, the inductance values can be readily adjusted.

Compared with the Class-E power amplifier scheme, the inverse Class-E power amplifier can use a lower inductance value, which can improve the linearity of the output voltage [75]. The components in the output matching circuit can be obtained using the characteristics of the inverse Class-E power amplifier under ideal conditions [76]; therefore, theoretical values were obtained. Subsequently, some adjustments of these component values were required for the ultrasound transducers to achieve high linearity. The two inductance values can be calculated using Equation (7). The target operating frequency (f*_c_*) and output power (P_out_) must be determined to select the two inductance values (L_4_ and L_5_).

(7) $$\left(L_{4}∥L_{5}\right)=\frac{V_{\mathrm{DD}}}{2\pi^{2}f_{c}P_{\mathrm{out}}},$$

where f*_c_* is the operating frequency of the designed inverse Class-E power amplifier.

Moreover, the value of C_4_ can be calculated using Equation (8), where the operating frequency and output power were determined. In Equation (8), the value of the inductance (L_6_) can be obtained, where the quality factor (Q_c_) depends on the bandwidth of the ultrasound transducer [77]. The typical bandwidth of ultrasound transducers is close to 50% of the operating frequency; therefore, the quality factor is close to 0.5 [78]. For imaging applications, the −6 dB bandwidth of the echo signal generated by the ultrasound transducer is supposed to be higher than 50% [79]. The conventional inverse Class-E power amplifier does not have a resistor (R_8_). However, a resistor was used in the ultrasound transducer load because it could reduce the overshoot problem [80]. In Equation (11), the capacitance (C_5_) can be calculated, whereas the inductance (L_6_) is determined using Equation (10).

(8) $$C_{4}=\frac{\pi \left(\pi^{2}-4\right)P_{\mathrm{out}}}{4\pi \left(\pi^{2}+4\right)f_{c}V_{\mathrm{DD}}}.$$

(9) $$R_{8}=\frac{\left(\pi^{2}+4\right)V_{\mathrm{DD}}{}^{2}}{8P_{\mathrm{out}}}.$$

(10) $$L_{6}=\frac{R_{8}}{2{\pi f}_{c}Q_{c}}.$$

(11) $$C_{5}=\frac{1}{4\pi^{2}f_{c}L_{6}}.$$

However, the calculated values of the output matching network in the inverse Class-E power amplifier cannot be directly applied because the values are theoretically obtained based on the highest power efficiency. Therefore, the values of the output matching network were adjusted to comprise the performance of the linearity and efficiency together for ultrasound transducers. Because the voltage gain difference could be obtained as less than 1 dB, a linear power amplifier could be achieved accordingly.

The linearity analysis of the power amplifier under high-voltage or high-power operating conditions does not provide accurate results, as presented in the existing studies [63,64]. In the simulation library, components, such as power resistors, electrostatic capacitors, variable resistors, and choke inductors, were not provided by manufacturers. Therefore, the experimental results of the designed Class-E power amplifier are provided for design guidance.

## 4. Results and Discussion

**Figure 5:**
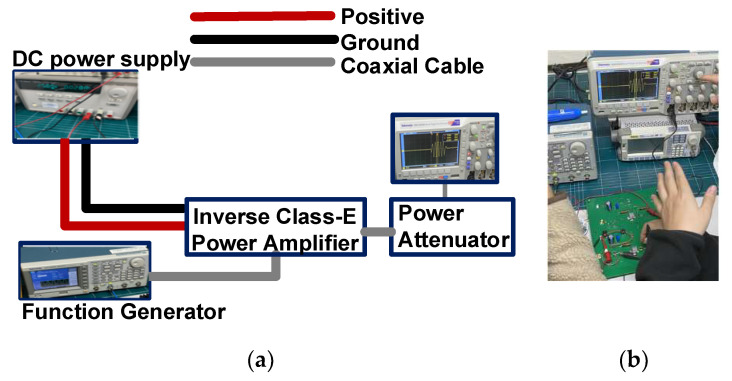
(**a**) Measurement setup and (**b**) photo for the performance of the designed inverse Class-E power amplifier.

Figure 5a,b illustrate the measurement setup and photo of the output voltage versus input voltage, output voltage versus frequency, power-added efficiency (PAE) versus input voltage, and PAE versus input frequency of the designed Class-E power amplifier. For acoustic stimulation/neuromodulation applications, the output voltage is the source parameter; therefore, the output voltage versus the input voltage was measured. A DC power supply (E3631A, Agilent Technology, San Jose, CA, USA) was used to apply a DC voltage to the designed inverse Class-E power amplifier. A function waveform generator (AFG3252C, Tektronix Inc., Beaverton, OR, USA) was used to generate multi-cycle sinusoidal signals. The amplified signals from the designed inverse Class-E power amplifier were attenuated using a power attenuator to protect the oscilloscope (MDO4104C, Tektronix Inc.).

**Figure 6:**
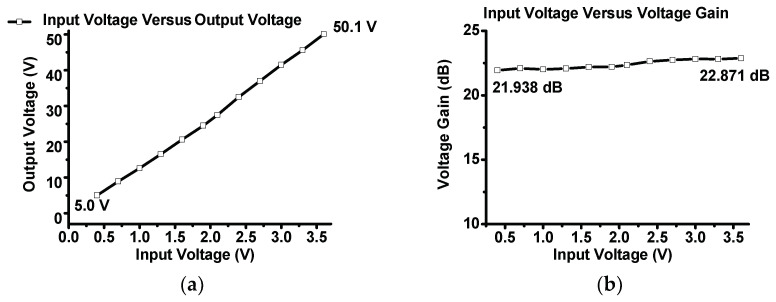

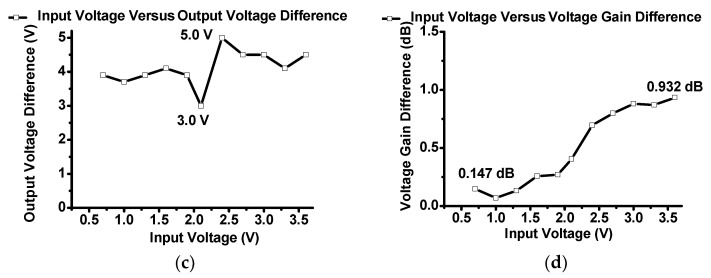
The experimental (**a**) output voltage, (**b**) voltage gain, (**c**) output voltage difference, and (**d**) voltage gain difference versus input voltage of the inverse Class-E power amplifier.

Figure 6a,b show the measured output voltage or voltage gain versus the input voltage of the designed inverse Class-E power amplifier. The function generator yielded 10-MHz input voltage signals in the 0.4–3.6 V range, with increments of 0.3 V. The voltage gain and voltage gain difference were calculated using Equations (12) and (13), respectively, as follows:

(12) $$\mathrm{Voltage}\;\mathrm{Gain}=20{\mathrm{Log}}_{10}\frac{V_{\mathrm{out}}}{V_{\mathrm{in}}},$$

(13) $$\mathrm{Voltage}\;\mathrm{Gain}\;\mathrm{Difference}=\mathrm{Voltage}\;\mathrm{Gain}\;\left(\mathrm{next}\right)-\mathrm{Voltage}\;\mathrm{Gain}\;\left(\mathrm{previous}\right),$$

where V_out_ and V_in_ are the measured output and input voltages, respectively. “Voltage Gain (next)” and “Voltage Gain (previous)” are the current calculated voltage gain and previously calculated voltage gain of the inverse Class-E power amplifier, respectively.

In Figure 6a, the lowest output voltage is 5.0 V at an input voltage of 0.4 V, and the highest output voltage is 50.1 V at an input voltage of 3.6 V. In Figure 6b, the lowest and highest voltage gains were 21.93 and 22.87 dB, respectively. The highest voltage gain difference is less than 1 dB (0.932 dB). In addition, Equations (7) and (8) were used to adjust the values of the output matching network components several times to reduce the voltage gain differences between the lowest and highest voltage gains.

Figure 6c shows the measured output voltage difference versus the input voltage between the adjacent input voltages. As shown in Figure 6c, the input voltage was increased at intervals of 0.3 V, and the output voltage difference was in the range of 3.0–5.0 V. Figure 6d shows the voltage gain difference versus input voltage between the lowest and highest voltage gains of the designed inverse Class-E power amplifier. Therefore, the measured data show that the designed inverse Class-E power amplifier is a linear power amplifier within the desired input voltage ranges.

**Figure 7:**
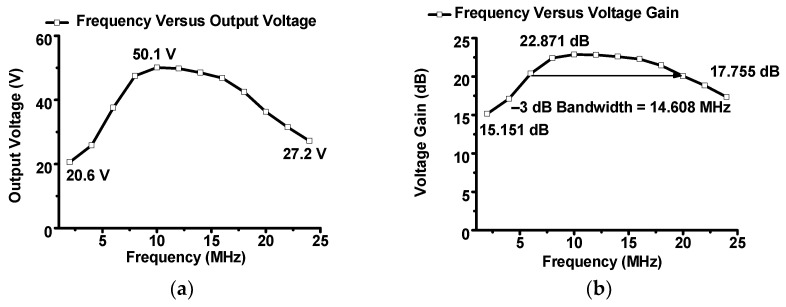

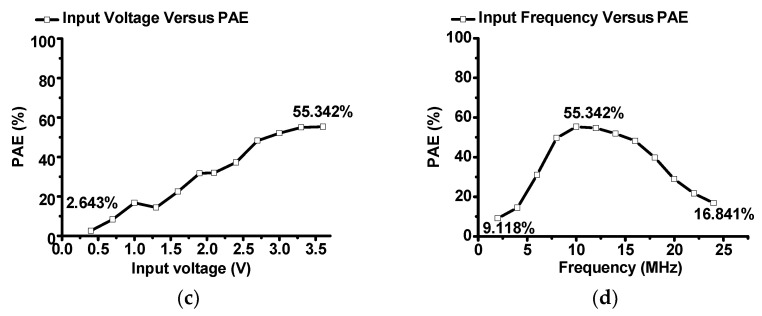
Measured (**a**) output voltage and (**b**) voltage gain versus input frequency, (**c**) PAE versus input voltage, and (**d**) PAE versus input frequency of the inverse Class-E power amplifier.

Figure 7a,b show the measured output voltage and voltage gain versus the input frequency, respectively. In Figure 7a, the output voltages were 20.6, 50.1, and 27.2 V at 2, 10, and 24 MHz, respectively. In Figure 7b, the highest voltage gain was 22.871 dB at 10 MHz, and the lowest voltage gain was 15.151 dB at 2 MHz. The −3 dB bandwidth was 14.608 MHz; thus, the designed amplifier can be used in the range of 4–20 MHz. The calculated PAE is expressed in Equation (14) because an inverse Class-E power amplifier is a nonlinear power amplifier that is concerned with power efficiency. The PAE was measured by varying the input voltage and frequency to estimate the power efficiency. This is because the linearity and PAE of the inverse Class-E power amplifier also have a trade-off relationship [81]. In communication systems, a high PAE is desirable in order to sacrifice linearity [82]. However, in some ultrasound applications, high linearity is one of the key factors. Thus, the linearity and PAE performance of the designed inverse Class-E power amplifier must be compromised. Even though a highly linear inverse Class-E power amplifier was obtained, the power efficiency must be considered. PAE is expressed as

(14) $$\mathrm{PAE}\;\left(\%\right)=\left(\frac{P_{\mathrm{OUT}}-P_{\mathrm{IN}}}{P_{\mathrm{DC}}}\right)\times 100,$$

where P_OUT_, P_IN_, and P_DC_ are the measured output, input, and DC power consumption, respectively.

Figure 7c,d show the measured PAE versus input voltage and frequency. In Figure 7c, the input voltage was in the range of 0.4–3.6 V. The lowest PAE was 2.643% at the input voltage and frequency of 0.4 V and 10 MHz, respectively, whereas the highest PAE was 55.342% at the input voltage and frequency of 3.6 V and 10 MHz, respectively. As expressed in Equations (7) and (8), a larger capacitance value of C_4_ could be obtained; however, lesser inductance values of L_4_ and L_5_ could be obtained. In Figure 7d, the input voltage was set to 3.6 V, and the input frequency varied in the range of 2–24 MHz. The measured lowest PAE was 9.118% at 2 MHz, and the highest PAE was 55.342% at 10 MHz.

**Figure 8:**
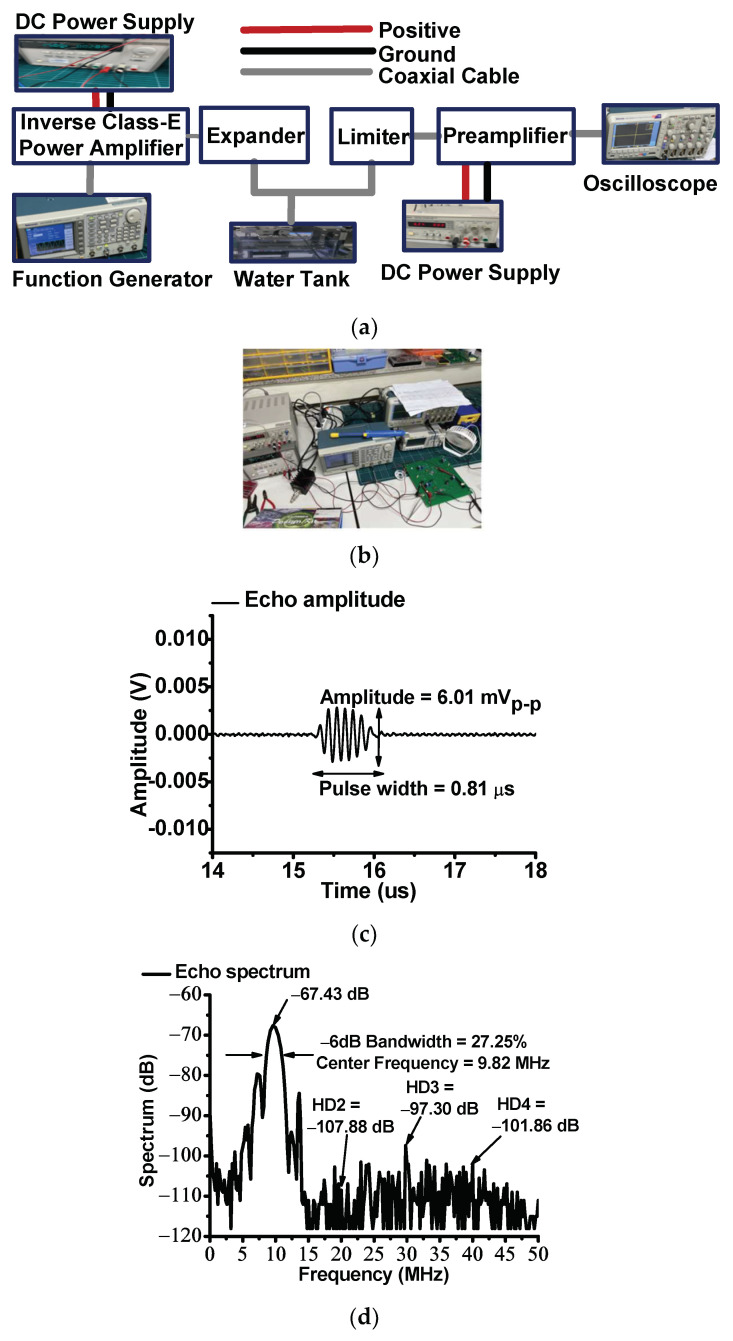
(**a**) Measurement setup and (**b**) photo of the pulse-echo response with designed inverse Class-E power amplifier and ultrasound transducer. The measured echo signal amplitude and spectrum in the (**c**) time and (**d**) frequency domains when using the designed inverse Class-E power amplifier and ultrasound transducer.

Figure 8a,b show the test setup and photo of the pulse-echo response when using the designed inverse Class-E power amplifier and ultrasound transducer, respectively. The test setup is a typical pulse-echo response; hence, the power amplifier and expander were used to drive the ultrasound transducer [83,84,85]. The power amplifier was located to drive the transducer before the expander circuit [86]. The expander is composed of a series-connected single diode pair [87]. The limiter, composed of a resistor of 50-Ω parallel with a single diode pair, was used to protect the preamplifier and oscilloscope against overshoot voltages [88]. The preamplifier was a voltage amplifier (AD8001, Analog Devices Inc., Santa Clara, CA, USA). The weak echo signal amplitude, received by the ultrasound transducer, was amplified by the preamplifier; the signal was displayed using the oscilloscope [89]. The spectrum data could be observed in the oscilloscope using a fast Fourier transform function. The pulse-echo response was performed because the designed inverse Class-E power amplifier could measure the pulse duration and center frequency.

The pulse width was obtained by calculating the peak-to-peak amplitude variance of 1% from the highest peak-to-peak amplitude of the echo signal [90,91]. The center frequency and −6 dB bandwidth of the echo signal were obtained by calculating the spectrum data at the left and right points −6 dB below the highest spectrum data, as presented in [92,93]. The pulse width, center frequency, and −6 dB bandwidth are expressed as follows:

(15) $$\mathrm{Pulse}\;\mathrm{width}\;\left(\mu S\right)=\mathrm{time}\left(\mathrm{right}\right)-\mathrm{time}\left(\mathrm{left}\right),$$

(16) $$\mathrm{Center}\;\mathrm{frequency}\;\left(\mathrm{MHz}\right)=\frac{\left(f_{c1}+f_{c2}\right)}{2},$$

(17) $$-6\;\mathrm{dB}\;\mathrm{bandwidth}\;\left(\%\right)=\left(\frac{\left(f_{c2}-f_{c1}\right)}{\mathrm{center}\;\mathrm{Frequency}}\right)\times 100,$$

where time (right) and time (left) in Equation (15) are the measured points located at the right and left sides from 1% of the peak-to-peak amplitude, respectively, and f_*c*1_ and f_*c*2_ in Equations (16) and (17) are the measured frequency points located at the left and right sides, respectively, which are 6 dB below the maximum spectrum data.

As shown in Figure 8c, the measured amplitude and pulse width of the echo signal was 6.01 mV_p-p_ and 0.81 μsec, respectively. In particular, the original pulse width of the input pulse waveform was 0.3 μs. Figure 8d shows the spectrum of the echo signal. As shown in Figure 8d, the center frequency and −6 dB bandwidth of the echo signal was 9.82 MHz and 29.93%, respectively. The second, third, and fourth harmonic distortion values (HD2, HD3, and HD4) are −107.88, −97.30, and −101.86 dB, respectively. In addition, these measured data confirmed that the designed inverse Class-E power amplifier slightly altered the operating frequency of the ultrasound transducer.

Table 1 summarizes the performance of the designed inverse Class-E power amplifier, including the pulse-echo response data. The output voltage, voltage gain, and PAE were measured at the input frequency and voltage values of 10 MHz and 3.6 V, respectively. The pulse-echo response was performed using an ultrasound transducer.

Table 2 summarizes the developed power amplifiers for ultrasound applications with the designed inverse Class-E power amplifier. Table 2 reveals that the designed inverse Class-E power amplifier exhibited a relatively good PAE at higher-frequency operating conditions and a lower harmonic distortion performance than those of the previous study, as shown in Table 1. Compared with previous work, the designed inverse Class-E power amplifier could show a relatively good power efficiency of 55.342% and a proper output voltage of 50.1 V at 10 MHz. In [94], the HD2, when using the Class-A power amplifier, was achieved as −45.61 dB. In [95], the HD2 of −21.75 dB was achieved. In [53], the HD2, when using the Class-AB power amplifier, was achieved as −50 dB. For the inverse Class-E power amplifier, the HD2 was −107.88 dB, which is less than −40 dB from the spectrum data at the fundamental frequency (–67.43 dB). Compared with Class-A and Class-AB power amplifiers, the designed inverse Class-E power amplifier achieved comparable harmonic distortion.

## 5. Conclusions

Ultrasound imaging and acoustic stimulation have been used in ultrasound applications. The desirable characteristics of the power amplifiers are their high linearity and relatively high power efficiency. However, linear power amplifiers, such as Class-A power amplifiers, have high linearity but low efficiency, whereas nonlinear power amplifiers have low linearity but high efficiency. Therefore, in this study, an inverse Class-E power amplifier with high linearity and relatively high efficiency was designed for ultrasound applications.

The proposed inverse Class-E amplifier was redesigned to drive an ultrasound transducer. Class-A power amplifiers simultaneously yield high linearity and low signal distortions; however, they generate high amounts of unwanted heat. Therefore, a Class-A power amplifier with a long-duration operation might affect the performance of the ultrasound transducer in acoustic stimulation applications. Therefore, nonlinear power amplifiers, such as the Class-C, Class-D, and Class-E power amplifiers, could be alternative candidates. Such power amplifiers exhibit a reasonable power efficiency; however, they have high signal distortions. Therefore, a new inverse Class-E power amplifier with relatively high linearity was designed for the ultrasound transducer.

Moreover, the linearity and PAE were measured to verify the performance of the designed inverse Class-E power amplifier. At an input frequency and voltage of 10 MHz and 3.6 V, respectively, the measured output voltage and voltage gain were 50.1 V and 22.871 dB, respectively. The voltage gain difference in the voltage range of 0.4–3.6 V was 0.932 dB, which was less than the 1 dB range. The measured PAE at an input frequency and voltage of 10 MHz and 3.6 V was 55.342%. Therefore, the designed amplifier exhibited reasonable linearity.

The pulse-echo response with a 10 MHz ultrasound transducer was performed to further verify the capability of the designed inverse Class-E power amplifier. The measured pulse width of the echo signal was 0.81 μs. In the spectrum data, the measured second, third, and fourth harmonic distortion values were −107.88, −97.30, and −101.86 dB, respectively. The center frequency of the echo signals was 9.82 MHz. These measured data indicated that the designed inverse Class-E power amplifier did not significantly affect the operating frequency of the ultrasound transducer. Consequently, the designed inverse Class-E power amplifier could be one of the candidates to improve the acoustic trapping performance.

In the future, the inverse Class-E power amplifier will be applied in ultrasound neuromodulation and stimulation applications after packaging the power amplifier. In ultrasound neuromodulation applications, the power amplifier must be operated at a high power efficiency, owing to its long duration; therefore, a highly efficient power-supply attached power amplifier design must be introduced. In ultrasound stimulation applications, the operating frequency of the power amplifier is higher than several MHz, such that high linear signal generation is important because of the high harmonic signal distortions. Therefore, a harmonic reduction filter scheme could be added after the output matching circuit.

## Figures and Tables

**Table 1:** Performance summary of the designed inverse Class-E power amplifier.

Output Voltage	Voltage Gain	PAE	Amplitude	Pulse Width	Center Frequency	−6 dB Bandwidth	HD2	HD3	HD4
50.1 V	22.871 dB	55.342%	6.01 mV_p-p_	0.81 μs	9.82 MHz	27.25%	−107.88 dB	−97.30 dB	−101.86 dB

**Table 2:** Summary of developed amplifiers for ultrasound applications.

	Class Type	Voltage Gain	Output Voltage or Output Power	−3 dB Bandwidth or Center Frequency	Harmonic Distortion	Efficiency	Application
[51]	Class-A	42 dB	60 V_p-p_				Ultrasound Imaging
[52]	Class-B	40.9 dB	90 V_p-p_	6.5 MHz			Ultrasound Imaging Transmitter
[53]	Class-AB		200 V_p-p_		−50 dB	30%	Ultrasound Transmitter
[54]	Class-AB		1,400 V	20.046 kHz			Sandwiched Piezoelectric Transducer
[55]	Class-AB		60 V_p-p_	15 MHz			High-power Piezoelectric Transducer
[56]	Class-D		2 kW	100 kHz			Power Piezoelectric Load
[57]	Class-D		125 V	3.6 kHz		95%	Dielectric Elastomer Transducer
[58]	Class-DE		2 kW	100 kHz			Acoustic Cavitation Reactor
[59]	Class-E		133.3 mW	41.27 kHz			Inductive Piezoelectric Transducer
[60]	Class-E		39.7 W	41.27 kHz			Langevin Transducer
This	Inverse Class-E	22.871 dB	50.1 V	9.82 MHz	–107.88 dB	55.342%	Ultrasound Transducer

## Data Availability

Data presented in this study is included in this article.
